# Association of neuropeptide Y promoter polymorphism (*rs16147*) with perceived stress and cardiac vagal outflow in humans

**DOI:** 10.1038/srep31683

**Published:** 2016-08-16

**Authors:** Hsin-An Chang, Wen-Hui Fang, Tieh-Ching Chang, San-Yuan Huang, Chuan-Chia Chang

**Affiliations:** 1 Department of Psychiatry, Tri-Service General Hospital, National Defense Medical Center, Taipei, Taiwan; 2 Department of Family and Community Medicine, Tri-Service General Hospital, National Defense Medical Center, Taipei, Taiwan; 3 Graduate Institute of Medical Sciences, National Defense Medical Center, Taipei, Taiwan

## Abstract

Neuropeptide Y (NPY) is involved in resilience to stress, and higher vagal (parasympathetic) activity has been associated with greater stress resilience. Thus, we examined whether *rs16147*, a functional promoter polymorphism (C>T) of the *NPY* gene, could influence vagal tone during chronic high stress levels. *NPY* genotyping, chronic psychological stress level measurement (using the Perceived Stress Scale [PSS]), cardiac autonomic function assessment (using short-term heart rate variability [HRV]) were performed in 1123 healthy, drug-free Han Chinese participants who were divided into low- and high-PSS groups. In the high-PSS group (*n* = 522), the root mean square of successive heartbeat interval differences and high frequency power (both HRV indices of parasympathetic activity) were significantly increased in T/T homozygotes compared to C/C homozygotes. However, no significant between-genotype difference was found in any HRV variable in the low-PSS group (*n* = 601). Our results are the first to demonstrate that functional *NPY* variation alters chronic stress-related vagal control, suggesting a potential parasympathetic role for *NPY* gene in stress regulation.

High levels of prolonged stress may lead to physiological and various psychological problems^1^ but do not affect everyone similarly. Resilient people have adaptive physiological and psychological responses and are not weighed down by adversity, while non-resilient people exhibit inappropriate responses that increase their susceptibility to disease^2^. Recent studies of heart rate variability (HRV), a noninvasive electrocardiographic (ECG) method to measure autonomic nervous system (ANS) activity^3^, have implicated ANS function in the stress response.

The cognitive and emotional responses of people with higher cardiac vagal (parasympathetic) tone tend to be more adaptive to a variety of stressors^4^,^5^,^6^. Higher vagal control further reduces the later development of internalizing psychopathology in response to chronic adversity^7^,^8^. In addition, higher vagal tone also predicts better cardiovascular recovery from psychosocial stress^9^,^10^. Conversely, decreased vagal control is associated with impaired post-stress recovery of cardiovascular, endocrine, and immune markers; predicts future stress-related mental disorders (e.g., anxiety and depressive disorders), and predisposes to cardiovascular risk factors such as obesity, hypertension, and diabetes^8^,^11^,^12^. Differences in vagal tone under high chronic stress conditions have therefore been considered a contributing factor to individual differences in resilience to stress^7^,^13^,^14^. However, we know little about the underlying molecular mechanisms responsible for individual differences in vagal control during prolonged high stress.

The 36-amino acid neuropeptide Y (NPY) is released during both acute and chronic stress^15^, widely distributed in the brain and ANS^16^, and an influential mediator of stress resilience. Animal models and pharmacological studies have repeatedly revealed the anxiolytic and antidepressant-like properties of NPY in stress-induced behaviors^17^,^18^,^19^,^20^. Likewise, human studies have demonstrated that soldiers with higher levels of NPY following interrogation displayed lower psychological distress^21^. Furthermore, higher NPY is positively associated with feelings of dominance and self-confidence, and superior performance under interrogation stress^22^,^23^. Of note, experimental research has also shown the involvement of the NPY system in synaptic transmission of the ANS, particularly the parasympathetic nervous system^24^,^25^.

A single nucleotide polymorphism (SNP *rs16147*: C-399T) in the promoter region of *NPY* gene has recently been shown to alter *NPY* expression *in vitro*, with -399C allele reducing the expression of *NPY* gene^26^. Previous studies have reported that the *NPY* -399C allele is associated with increased bilateral amygdala activation in response to threat-related fascial expression in both healthy humans and depressed patients^26^,^27^. In addition, the *NPY rs16147* also modifies risk of post-disaster generalized anxiety disorder under conditions of hurricane exposure^28^. Thus, investigating the effect of *NPY rs16147* polymorphism on HRV during long periods of high stress is crucial for understanding the mechanisms underlying chronic stress-related vagal control and may provide potential insights into NPY’s role in stress resilience.

Several non-genetic factors (age, gender, smoking status, physical activity, physical position, medications, and psychiatric and medical morbidities) may influence HRV^29^. Studying medication-free healthy subjects in a well-controlled manner can therefore attenuate these confounding influences to reveal more precisely the effect of the *NPY* gene on stress regulation of vagal activity. Using HRV measures in a large cohort of healthy drug-free Han Chinese subjects, the current study tested the hypothesis that the promoter variant (*rs16147*: C-399T) of *NPY* has an effect on cardiac vagal control during high-level chronic stress and whether the association remains significant even after adjusting for relevant confounders.

## Results

### Sample characteristics in *NPY* genotype subgroups of low- and high-stress participants

The *NPY* genotype distribution in this cohort was in Hardy-Weinberg equilibrium. Using a median split approach, we divided participants into those with high Perceived Stress Scale (PSS) score (*n* = 522; mean PSS score: 27.23 ± 4.38; range: 22–45) and those with low PSS score (*n* = 601; mean PSS score: 15.19 ± 4.60; range: 0–21). The demographic and clinical data for the participants with different *NPY* genotypes in both groups are listed in Table 1. In the low-PSS group, the *NPY* genotype subgroups did not differ in demographic data or clinical characteristics, e.g., gender, systolic (SBP) and diastolic blood pressure (DBP), serum metabolic profiles, and Beck Anxiety Inventory (BAI) scores. However, the *NPY* genotype subgroups of high-PSS participants differed significantly in heart rate (HR), SBP, and DBP (*p* = 0.017, 0.021, and 0.002, respectively). T/T genotype carriers had significantly lower values of HR, SBP, and DBP than C/C genotype subjects (all *p* < 0.05), while C/T heterozygotes and T/T homozygotes had similar HR and blood pressure.

### Non-genetic factors associated with HRV indices, stratified by PSS score

Associations between HRV measures and potential confounding variables are summarized separately according to PSS score (Table 2). In the high-PSS group, women (compared to men) had significantly reduced low frequency power (LF), high frequency power (HF), ratio of LF to HF (LF/HF), and root mean square of successive heartbeat interval differences (RMSSD). Habitually physically active participants had significantly higher HF and RMSSD. Age, BAI score, and serum metabolic parameters were negatively correlated with HRV indices, including HF and RMSSD. For the low-PSS group, age, gender, body mass index (BMI), habitual physical activity, and serum metabolic parameters were related to at least one index of HRV.

### Association of *NPY* genotypes with HRV indices in the low- and high-stress groups

**Figure 1:**
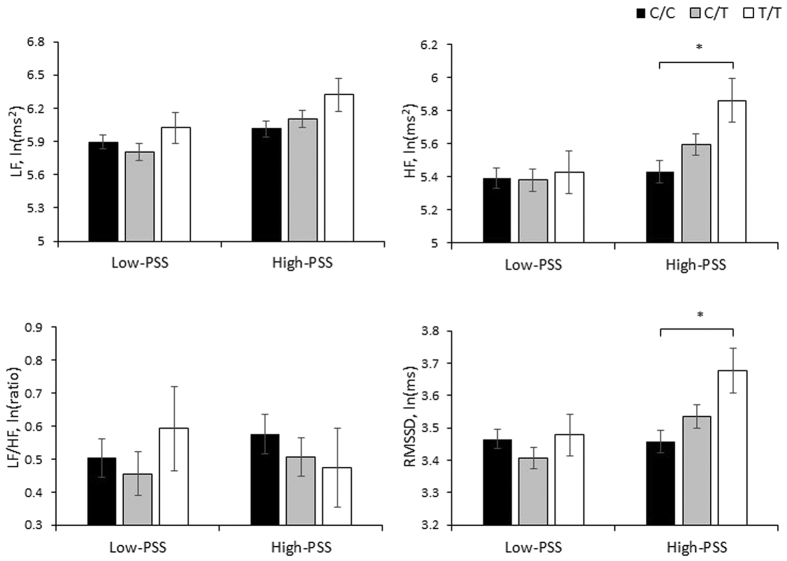
Unadjusted effects of the *NPY* genotype on indices of heart rate variability, stratified by PSS score. LF, low frequency power; HF, high frequency power; LF/HF, ratio of LF to HF; RMSSD, the root mean square of successive heartbeat interval differences. PSS, Perceived Stress Scale; Low- and high-PSS were determined by a median split at PSS score 21. *p < 0.05.

The relationships of HRV indices to *NPY* genotype in both the low- and high-PSS groups are presented in Fig. 1. In the high-PSS group, three *NPY* genotypes differed in HF (F = 4.56, *p* = 0.011) and RMSSD (F = 4.15, *p* = 0.016). Participants bearing the T/T genotype displayed increased HF (*p* = 0.012) and RMSSD (*p* = 0.016) compared to C/C genotype carriers, but C/T heterozygous and T/T homozygous participants had similar HRV indices. Furthermore, the *NPY* genotype had no effect on HRV measures in the low-PSS group.

Variables associated with HRV were used as covariates in ANCOVA models, with HRV indices as dependent variables. After adjusting for relevant confounders, including age, gender, BAI, habitual physical activity levels, and serum metabolic profiles, the aforementioned associations remained significant (Table 3).

**Figure 2:**
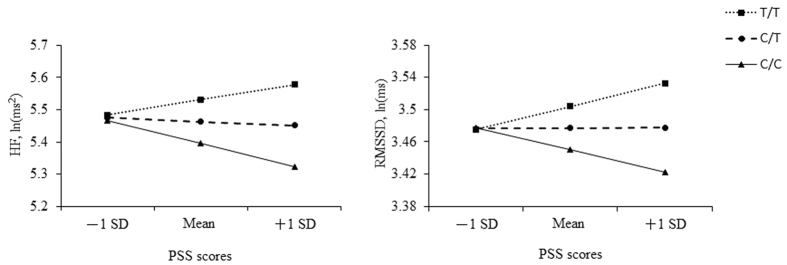
Moderation effects of PSS on the associations between *NPY* genotypes and vagus-mediated HRV. HF, high frequency power; RMSSD, the root mean square of successive heartbeat interval differences; SD: standard deviation; PSS, Perceived Stress Scale.

Furthermore, using the SPSS macro PROCESS^30^ to analyze the total sample (adjusting for age and gender), *NPY* genotypes had a significant main effect on HF (*B* = 0.10, *SE* = 0.04, *t* = 2.45, *p* = 0.014) and a marginally significant effect on RMSSD (*B* = 0.04, *SE* = 0.02, *t *= 1.90, *p* = 0.058), but PSS scores were not. The interaction term (product of PSS scores and *NPY* genotypes), however, was a significant predictor of HF (*B* = 0.01, *SE* = 0.01, *t* = 2.12, *p* = 0.034) and RMSSD (*B* = 0.01, *SE* < 0.01, *t* = 2.03, *p* = 0.042). Two copies of -399T allele were significantly associated with greater HF at relatively higher PSS [(mean and +1 standard deviation (SD)] levels (*B* = 0.10, *SE* = 0.04, *t* = 2.15, *p* = 0.014; *B* = 0.19, *SE* = 0.06, *t* = 3.30, *p* = 0.001, respectively). Similarly, the effects were also found in RMSSD [(for mean: *B* = 0.04, *SE* = 0.02, *t* = 1.90, *p* = 0.058 (marginally significant); for +1 SD: *B* = 0.08, *SE* = 0.03, *t* = 2.67, *p* = 0.008)] (Fig. 2).

## Discussion

The present study found that the functional *NPY* promoter polymorphism (*rs16147*) interacts with chronic stress to influence autonomic control. Under high chronic stress, vagal activity is elevated (i.e., HF and RMSSD are significantly higher) in T/T genotype carriers than C/C genotype carriers. However, *NPY* genotype does not affect vagus-mediated HRV measures in subjects with chronic low level stress. After adjusting for relevant covariates, the association of *NPY* with vagal indices of HRV remains significant. Furthermore, moderation analyses also confirm the moderating role of PSS level on the association between *NPY* genotypes and HF and RMSSD. To our knowledge, this is the first study to investigate the role of a functional *NPY* genetic polymorphism in human vagal regulation during chronic psychological stress.

Evidence suggests that NPY may modulate ANS regulation of stress reactivity. Histological studies reveal the presence of NPY receptors (Y1, Y2, Y4, and Y5) in neurons and satellite cells of parasympathetic ganglia^31^. Research in animals has demonstrated that NPY blunts elevations in blood pressure and HR following exposure to social stress^32^, and that NPY pretreatment can block chronic stress-induced baroreflex hypersensitivity^33^. Furthermore, the synaptic release of acetylcholine, a critical neurotransmitter of the parasympathetic nervous system, is reported to be affected by NPY under laboratory stress^34^. Heritability studies have already demonstrated that genetics contribute substantially to variance in vagus-mediated HRV^35^. The findings of the present study conducted in human volunteers complement those of previous studies and show that the functional *NPY* genetic polymorphism plays a key role in stress regulation of autonomic vagal activity.

High levels of vagal tone have been considered a sign of autonomic flexibility, the capability of the parasympathetic nervous system to generate adequate responses to environmental stress by modifying HR, respiration, and arousal^5^,^14^. In contrast, decreases in vagal modulation may predict mismatches between environmental challenges and cardiac (re)-activity, thus increasing vulnerability to stress-related cardiovascular diseases (CVDs) such as angina pectoris, coronary heart disease, myocardial infarction, or congestive heart failure^36^,^37^. Recent studies have shown the involvement of *NPY* system polymorphisms in CVDs^38^,^39^, for example, the involvement of the *rs16147* -399C allele in the development of early-onset atherosclerosis^40^. Taken together, our findings showing that reduced cardiac vagal control in chronically high stressed individuals with the C/C genotype of *NPY* may suggest an underlying parasympathetic role for *NPY* gene in susceptibility to CVDs. Indeed, even in the present healthy cohort, high chronically stressed subjects with the C/C genotype already exhibited faster HR and higher SBP and DBP. A prospective study examining the impact of *NPY* variant-associated vagal decline on the incidence of stress-related CVDs is warranted.

Two studies identified the C/C genotype of *NPY rs16147* as a risk factor for stress-related psychopathology including anxiety and depression^26^,^41^, while another study failed to detect any effect of the *NPY* variant on anxiety or depression disorders^42^. Thus, an analysis of HRV using quantitative indices of ANS function^43^ to explain the physiologic role of the studied *NPY* polymorphism under chronic high stress conditions may complement analyses using conventional self-report questionnaire approaches, which often cannot effectively separate one phenotype from another^44^. Since reduced vagal activity is associated with anxiety and depression^6^,^45^, and the NPY system is implicated in stress-related psychiatric disorders^16^, the results here raise the possibility that the *NPY* C/C genotype with chronic stress-related low vagal activity increases the risk of developing stress-related psychopathology in the long run.

Investigating stress responses at multiple phenotypic levels, including not only self-report psychological measurements, but also measurements of ANS functions, could help to delineate the underlying mechanism of stress resilience^46^. As mentioned in the Introduction, higher levels of NPY and greater cardiac vagal control have both been associated with resilience to stress. It is noteworthy that the -399C allele (*rs16147*) reduces expression of *NPY* gene *in vitro* (i.e., decreases NPY mRNA levels by 30%)^26^. Therefore, the present result that, under chronic high stress, cardiac vagal tone is higher in “high-expression” T/T genotype carriers than C/C genotype carriers may suggest a potential parasympathetic pathway involving NPY in stress resilience. Furthermore, our finding also helps to explain, at least partly, why people differ in their resilience to stress.

Lastly, several non-genetic confounders may have effects on the vagus-mediated HRV. Different methods of data acquisition have often elicited inconsistent HRV results^29^. Nonetheless, our study has carefully controlled for confounding factors. The participants here were also all drug-free and had received medical health check-ups and structured psychiatric evaluations to exclude illnesses. Moreover, as ethnic stratification of study samples may reset population HRV patterns^47^, all subjects in the current study were unrelated Han Chinese, and recruited from a population pool in Taiwan that is known to be genetically homogeneous^48^. Thus, our study may precisely exhibit the *NPY* gene’s effect on autonomic stress regulation, without ethnic stratification bias. Collectively, these observations suggest that false-positive results are less likely.

Several limitations should be considered in the present study. The study only used cross-sectional data. Therefore, the long-term influence of the *NPY* variant on autonomic stress regulation cannot be directly deduced. In addition, the reliability of using 5-min HRV recordings should be a concern. However, short-term HRV recordings have been shown to be reliable particularly in healthy individuals at rest^49^. Another limitation is that we did not control for the respiratory rate, which has been shown to influence parasympathetic measures of HRV in clinical settings^29^. Fortunately, this may not have affected our results because differences in HF between metronome-guided and spontaneous breathing are extremely small in healthy subjects^50^. Furthermore, only a single *NPY* variant was investigated. Future work should consider other genetic variations and other promising candidate genes, such as the serotonin transporter gene, to determine their additive and interactive effects. Our study failed to provide the information concerning the menstrual cycles of our female participants inasmuch as HRV has been shown to fluctuate between phases of the female menstrual cycle^51^. The levels of NPY are mainly determined by *NPY* gene expression, and the T/T (high expression) genotype may therefore have higher serum NPY levels contributing to related phenotypes^26^. However, serum NPY levels were not measured in the present study. Future studies analyzing not only *NPY* gene but also serum NPY levels may further strength understanding of the relationship between *NPY* variation and vagal control. Lastly, unequal subgroup sample size with relatively small numbers of the *NPY* T/T genotype carriers (*n* = 64 and 58, respectively in the Low- and High-PSS groups) may increase the type I error, even though the current sample composed of 1123 participants. Since this is the first study to look at this matter, studies replicated in independent, larger cohorts are necessary to corroborate the present results.

The current results provide initial evidence that the studied functional *NPY* polymorphism (*rs16147*) modulates vagal outflow to the heart during high chronic stress, suggesting a potential parasympathetic role for *NPY* gene in stress regulation. A follow-up study investigating the influence of the *NPY* polymorphism-associated vagal decline on the development of stress-related psychopathology and CVDs is warranted.

## Methods

### Subjects

The study cohort was recruited from volunteers who received annual health examinations at the Tri-Service General Hospital, a medical teaching hospital of the National Defense Medical Center in Taipei, Taiwan. All participants were unrelated ethnic Han Chinese. After an initial questionnaire evaluation, those taking any medication for at least 1 month before the study or those with a personal history of psychiatric illnesses, illegal substance dependence, medical diseases, or pregnancy were excluded. In all, 1321 Han Chinese adults were recruited. The demographic data collected included age, gender, BMI (kg/m^2^), weekly exercise level (0–2 times per week/≥3 times per week), and smoking status (yes/ no).

The study protocol was approved by the institutional review board of the Tri-Service General Hospital, which adhered to the guidelines of the Declaration of Helsinki, and all participants provided informed consent before the study.

### Measurement of chronic psychological stress

Chronic stress was assessed using the Chinese version of the PSS, which is a 14-item self-reported questionnaire that evaluates the degree to which individuals appraise their lives as unpredictable, uncontrollable, or overloaded during the last month^52^. Responses are rated on a frequency scale of 0 (never) to 4 (almost always). Scores can be used to infer relative stress levels or make within-group comparisons, with higher scores on the PSS indicating greater levels of psychological stress (range 0–56). The PSS is one of the most frequently and widely used tools to measure chronic stress^53^. For the stratified analysis conducted in this study, participants were divided based on a median split^54^ (median = 21) into low stress (PSS score ≤ 21) and high stress (PSS score > 21) groups.

### Assessment of psychiatric morbidity and anxiety and mood levels

The psychiatric morbidity of each enrolled participant was evaluated by a senior research assistant using the Chinese version of the Mini-International Neuropsychiatric Interview according to DSM-IV criteria^55^. Participants with psychiatric disorders were excluded, whereas the rest were further evaluated to determine their depression and anxiety status.

The Chinese version of the BAI, a self-rated 21-item inventory, was used to assess the intensity of anxiety over the past week^56^. Each item’s score ranged from 0 ‘not at all’ to 3 ‘severe’. The total scores ranged from 0–63, with higher scores indicating higher anxiety. The Chinese version of the Beck Depression Inventory-II (BDI), a 21-item questionnaire, was employed to assess the subjects’ self-reported levels of depression in the past two weeks^57^. Each item’s score ranged from 0 to 3, with 3 representing the most severe level (total score range, 0–63). Higher total score correlated with more severe depression. Both the Chinese BAI and BDI were highly valid and reliable^56^,^57^. Subjects with a higher than mild degree of anxiety (BAI > 15) or depression (BDI > 19) were also excluded.

### Measurement of medical conditions

Participants all received health check-ups, which included physical examination, biochemical analysis, and chest X-ray and ECG examinations. SBP and DBP were measured. Fasting plasma glucose, serum triglyceride, and total cholesterol levels were determined as described elsewhere^58^.

Subjects with cardiovascular diseases (e.g., arrhythmia and hypertension), metabolic disorders (e.g., hypertriglyceridemia, hypercholesterolemia, and diabetes mellitus), kidney or liver diseases, malignancy, neuropathy, or obesity (BMI ≥ 30 kg/m^2^) were excluded. The final study sample had 1123 healthy participants (541 men, 582 women; mean age: 38.31 ± 10.34).

### Assessment of heart rate variability

HRV was measured using an ECG analyzer (SA-3000P; Medicore Co., Ltd., Seoul, Korea) that acquired, stored, and processed ECG signals^59^. All of the subjects were examined in a quiet and temperature-controlled (22–24 °C) room after receiving psychometric evaluation. ECG monitoring was done in the morning (08:00–12:00 a.m.) to accommodate diurnal variations. After 15 min of sitting at rest, each participant underwent ECG recording in a sitting position for 5 min. The average HR (beats per minute) was derived from the R waves of the ECG.

Using time-domain and frequency-domain analyses^60^, the system analyzed changes in HR automatically. The time-domain index (RMSSD) reflected cardiac vagal activity. Power spectral analysis of changes in HR, using the fast-Fourier transformation, was then quantified into standard frequency-domain measurements, including LF (0.04–0.15 Hz), HF (0.15–0.4 Hz), and LF/HF^3^. The HF represented parasympathetic control of the HRV, whereas LF represented both vagal and sympathetic control of the HRV. The LF/HF ratio was considered by some investigators to reflect global sympathovagal balance or sympathetic modulation^3^. All the indices were logarithmically transformed to produce normalized distributions.

### SNP genotyping

Blood sampling was all performed one hour before the examination of HRV. For genotyping, DNA was isolated from blood samples using a QIAamp^®^ DNA blood kit and protocol based on the manufacturer’s instructions (Qiagen^®^, Valencia, CA). The quality of isolated genomic DNA was evaluated for each sample by agarose gel electrophoresis, while the quantity of DNA was measured by spectrophotometry (NanoDrop^®^, Wilmington, DE, USA). The studied *NPY rs16147* (C-399T) polymorphism was genotyped by the TaqMan^®^ 5′-exonuclease assay as described previously^41^ using a Prism 7900 system (Applied Biosystems^®^ Foster City, CA, USA).

### Statistical analysis

The allele and genotype frequencies of the *NPY* gene in participants stratified by PSS category were calculated and genotypic distribution was compared to values predicted from Hardy-Weinberg equilibrium. The χ^2^ Tests were used to compare categorical variables, and the ANOVA was used for continuous variables among the *NPY* genotype groups.

To control the non-genetic confounders, the associations between HRV indices and demographic/clinical variables (e.g., age, gender, cigarette smoking, and serum metabolic measurements) were tested. The Pearson correlation was used to test relationships between variables with normal distribution, whereas the Spearman correlation was used for variables with non-normal distribution. Variables correlated with HRV were then used as covariates in separate ANCOVA models evaluating the effects of genotype on each of the HRV indices. Bonferroni correction was applied to the post hoc testing. Additionally, we further analyzed the whole sample, controlling for age and sex, by using the SPSS macro PROCESS^30^ to test whether PSS scores did moderate the relationship between *NPY* genotypes and HRV indices. A *p* value < 0.05 (two-tailed) was considered statistically significant. All statistical analyses were done through IBM SPSS Statistics, Version 19.0.

Quanto software version 1.2.4^61^ was used for the power calculations. Both the high- and low-PSS cohorts (*n* = 522 and 601, respectively) had powers of 99.9% for determining a proportion of variance, *R*^2^ = 0.05, in HRV indices explained by *NPY* effects.

## Additional Information

**How to cite this article**: Chang, H.-A. *et al*. Association of neuropeptide Y promoter polymorphism (*rs16147*) with perceived stress and cardiac vagal outflow in humans. *Sci. Rep.* **6**, 31683; doi: 10.1038/srep31683 (2016).

## Figures and Tables

**Table 1:** Demographic data and clinical characteristics of participants stratified by *NPY* genotype and stress level.

Characteristics	aLow-PSS group (*n* = 601)			*P*^b^	aHigh-PSS group (*n* = 522)			*p*^b^
	C/C (*n* = 301)	C/T (*n* = 236)	T/T (*n* = 64)		C/C (*n* = 230)	C/T (*n* = 234)	T/T (*n* = 58)	
Age (year)	40.1 ± 10.5	41.6 ± 10.9	39.5 ± 10.7	0.20	35.3 ± 9.2	36.1 ± 9.3	35.2 ± 8.9	0.61
Female, *n* (%)	162 (53.8)	130 (55.1)	35 (54.7)	0.96	120 (52.2)	105 (44.9)	30 (51.7)	0.26
BMI (kg/m^2^)	22.6 ± 3.3	22.9 ± 3.2	22.5 ± 3.2	0.46	22.8 ± 3.4	22.9 ± 3.2	22.3 ± 3.7	0.49
Current smoker, *n* (%)	50 (17.4)	24.9 (13.6)	11 (17.5)	0.47	30 (13.6)	44 (19.8)	7 (13.7)	0.18
Weekly regular exercise				0.70				0.17
<3 times/ week, *n* (%)	241 (80.1)	193 (81.8)	54 (84.4)		206 (89.6)	200 (85.5)	47 (81.0)	
≥3 times/ week, *n* (%)	60 (19.9)	43 (18.2)	10 (15.6)		24 (10.4)	34 (14.5)	11 (19.0)	
Total cholesterol (mg/dl)	177.3 ± 27.4	181.1 ± 30.3	181.4 ± 29.9	0.25	174.6 ± 28.3	175.0 ± 28.0	173.9 ± 32.5	0.97
Triglyceride (mg/dl)	93.7 ± 39.6	93.0 ± 41.9	94.0 ± 44.0	0.98	93.6 ± 37.4	90.3 ± 37.2	93.2 ± 40.4	0.63
Fasting glucose (mg/dl)	87.6 ± 8.2	88.5 ± 8.5	85.9 ± 6.2	0.075	87.0 ± 7.9	87.2 ± 8.0	85.1 ± 7.46	0.19
PSS (scores)	15.4 ± 4.4	15.0 ± 4.8	14.9 ± 5.0	0.67	27.3 ± 4.0	27.1 ± 4.4	27.5 ± 5.6	0.84
BAI (scores)	2.84 ± 3.50	2.94 ± 3.68	3.98 ± 3.28	0.93	5.53 ± 4.71	5.53 ± 4.72	4.72 ± 4.38	0.47
BDI (scores)	3.69 ± 4.0	3.20 ± 3.4	2.83 ± 3.1	0.12	7.30 ± 5.0	7.47 ± 5.0	7.17 ± 5.4	0.90
Heart rate (beats/min)	70.7 ± 12.8	70.9 ± 11.3	70.9 ± 10.2	0.99	72.5 ± 10.2	70.7 ± 10.5	68.57 ± 10.3	**0.017**
SBP (mmHg)	112.2 ± 12.2	113.2 ± 13.1	109.6 ± 13.9	0.13	113.6 ± 12.8	112.4 ± 13.4	108.3 ± 11.4	**0.021**
DBP (mmHg)	73.7 ± 8.9	73.9 ± 9.1	71.0 ± 9.1	0.060	74.0 ± 9.0	72.2 ± 9.3	69.4 ± 8.3	**0.002**

^a^Low- and high-PSS groups were determined by a median split at PSS score 21.

^b^Comparison using ANOVA test (continuous variables) and χ^2^ test (categorical variables).

Continuous variables are reported as mean ± standard deviation; categorical variables are listed as number (percentage). Abbreviations: BAI, Beck Anxiety Inventory; BDI, Beck Depression Inventory-II; BMI, body mass index; PSS, Perceived Stress Scale; DBP, diastolic blood pressure; SBP, systolic blood pressure.

**Table 2:** Non-genetic potential confounding factors associated with HRV indices among participants stratified by PSS score.

	LF		HF		LF/ HF		RMSSD	
	Low-PSS	High-PSS	Low-PSS	High-PSS	Low-PSS	High-PSS	Low-PSS	High-PSS
Age	−0.45***	−0.45***	−0.41***	−0.38***	−0.09*	−0.17***	−0.39***	−0.37***
Gender (Women/men)	0.26***	0.26***	0.08*	0.15**	0.22***	0.20***	0.07	0.13**
BMI	0.02	0.01	−0.06	−0.01	0.08*	0.01	0.03	−0.06
Smoking status (No/ yes)	0.02	0.02	−0.003	−0.07	0.05	−0.05	0.05	0.08
Physical activity (Low/ high)	0.05	0.05	−0.03	0.10*	−0.09*	−0.06	0.03	0.15^***^
Total cholesterol	−0.13**	−0.15***	−0.11**	−0.07	−0.05	−0.09*	−0.16***	−0.11*
Triglyceride	−0.18**	−0.10*	−0.21***	−0.07	0.01	−0.03	−0.20***	−0.15**
Fasting glucose	−0.20***	−0.20***	−0.22***	−0.20***	−0.01	−0.04	−0.22***	−0.22***
BAI	−0.04	−0.15**	−0.06	−0.14**	0.01	−0.01	−0.06	−0.13^**^
BDI	−0.03	−0.05	0.01	−0.03	−0.06	−0.01	0.01	−0.04

Data are represented as correlation coefficient values.

First category in parenthesis is the reference group.

Physical activity levels were classified as 0–2 times per week (low)/ ≥3 times per week (high).

Low- and high-PSS groups were determined by a median split at a PSS score of 21.

Pearson correlation was used to evaluate the correlation between serum total cholesterol and HRV indices in the High-PSS group, and the others were analyzed by Spearman correlation.

Abbreviations: BAI, Beck Anxiety Inventory; BDI, Beck Depression-Inventory-II; BMI, body mass index; PSS, Perceived Stress Scale; HRV, heart rate variability; LF, low frequency power; HF, high frequency power; LF/HF, ratio of LF to HF; RMSSD, the root mean square of successive heartbeat interval differences.

*p < 0.05; **p < 0.01; ***p < 0.001.

**Table 3:** Adjusted means of heart rate variability (HRV) indices presented by *NPY* genotype and PSS score.

HRV index	^a^ Low-PSS group (*n* = 601)			*p*^†^	^a^ High-PSS group (*n* = 522)			*p*^†^	^b^ Post hoc comparisons
	C/C (*n* = 301)	C/T (*n* = 236)	T/T (*n* = 64)		C/C (*n* = 230)	C/T (*n* = 234)	T/T (*n* = 58)		
LF	5.87 ± 0.06	5.85 ± 0.07	5.96 ± 0.13	0.73	6.02 ± 0.07	6.11 ± 0.06	6.29 ± 0.13	0.16	
HF	5.38 ± 0.06	5.38 ± 0.06	5.36 ± 0.12	0.81	5.43 ± 0.06	5.61 ± 0.06	5.80 ± 0.12	**0.013**	C/C<T/T*
LF/HF	0.50 ± 0.06	0.46 ± 0.07	0.59 ± 0.12	0.67	0.59 ± 0.06	0.50 ± 0.06	0.48 ± 0.12	0.49	
RMSSD	3.46 ± 0.03	3.43 ± 0.03	3.44 ± 0.06	0.80	3.46 ± 0.03	3.55 ± 0.03	3.65 ± 0.06	**0.020**	C/C<T/T*

Data are presented as mean ± standard error.

^a^Low- and high-PSS groups were determined by a median split at PSS score 21.

^b^Bonferroni correction was applied to the post hoc testing.

†Adjusted for the covariates listed in Table 2 (variables associated with individual HRV indices were all entered as covariates in ANCOVA models with the corresponding index as a dependent variable).

Abbreviations: PSS, Perceived Stress Scale; LF, low frequency power [ln(ms^2^)]; HF, high frequency power [ln(ms^2^)]; LF/HF, ratio of LF to HF [ln(ratio)]; RMSSD, the root mean square of successive heartbeat interval differences [ln(ms)].

^*^p < 0.05.
